# A cautious approach for detecting and managing intercostal artery injury during CT-guided lung biopsy: a case report and literature review

**DOI:** 10.3389/fmed.2025.1709713

**Published:** 2025-11-17

**Authors:** Hao Zhang, Lin Li, Dong Lan, Jing Xu, Huan Leng, Xiang Liao, Chi Zhang

**Affiliations:** 1 Department of Radiology, Dianjiang People’s Hospital of Chongqing, Chongqing, China; 2 Department of Pharmacy, Dianjiang People’s Hospital of Chongqing, Chongqing, China; 3 Department of Medical Imaging, School of Basic Medical Sciences, Xinjiang Medical University, Urumqi, China

**Keywords:** intercostal artery injury, percutaneous transthoracic needle biopsy, coaxial needle, hemostasis, case report

## Abstract

Intercostal artery (ICA) injury during CT-guided percutaneous transthoracic needle biopsy (PTNB) is a rare but potentially severe complication. This case report describes a cautious approach for detecting and managing suspected ICA injury using a coaxial needle system. During PTNB in a 71-year-old male with lung cancer, ICA injury was detected through blood aspiration during needle withdrawal. Management involved immediate cessation of withdrawal, slight needle advancement for mechanical hemostasis, and serial monitoring, achieving hemostasis without requiring additional invasive interventions. Literature review of cases from 2018 to 2024 revealed that current management approaches vary based on severity, with a 30% mortality rate among reported cases. Most ICA injuries are detected post-procedurally rather than intraoperatively. Traditional interventions range from conservative treatment to transcatheter arterial embolization (TAE) or thoracotomy. This approach suggests a potential stepwise method for managing suspected ICA injury that might reduce complications and minimize the need for invasive interventions. As a single case report representing hypothesis-generating evidence, this observation requires further validation through collaborative experience from other operators when similar circumstances arise. The case demonstrates an exploratory approach for managing ICA injury during PTNB, particularly relevant in resource-limited settings where interventional radiology services may not be immediately available.

## 1 Introduction

Increased lung cancer screening recommendations using low-dose CT have amplified demand for percutaneous transthoracic needle biopsy (PTNB). While PTNB is generally safe, intercostal artery (ICA) injury remains a rare but potentially severe complication (1, 2). Given the substantial volume of PTNB procedures performed annually, even rare complications may affect a substantial number of patients. This case describes an exploratory approach for detecting and managing ICA injury during PTNB using a coaxial needle system. The Institutional Review Board (IRB) waived ethical approval requirements for this retrospective case report.

## 2 Case report

### 2.1 Patient information

A 71-year-old male presented with worsening cough, sputum production, and chest pain for 3 days. His medical history included chronic cough and sputum production for over 10 years. He had no history of allergies or bleeding disorders. Laboratory tests showed an elevated squamous cell carcinoma antigen level of 17.3 (reference value <3), with other tumor markers within normal ranges. Due to the patient’s severe emphysema with multiple large bullae, pulmonary function tests were not performed to avoid potential pneumothorax risk. Clinical assessment suggested severe obstructive ventilatory dysfunction.

### 2.2 Clinical findings and pre-procedure planning

**Figure 1:**
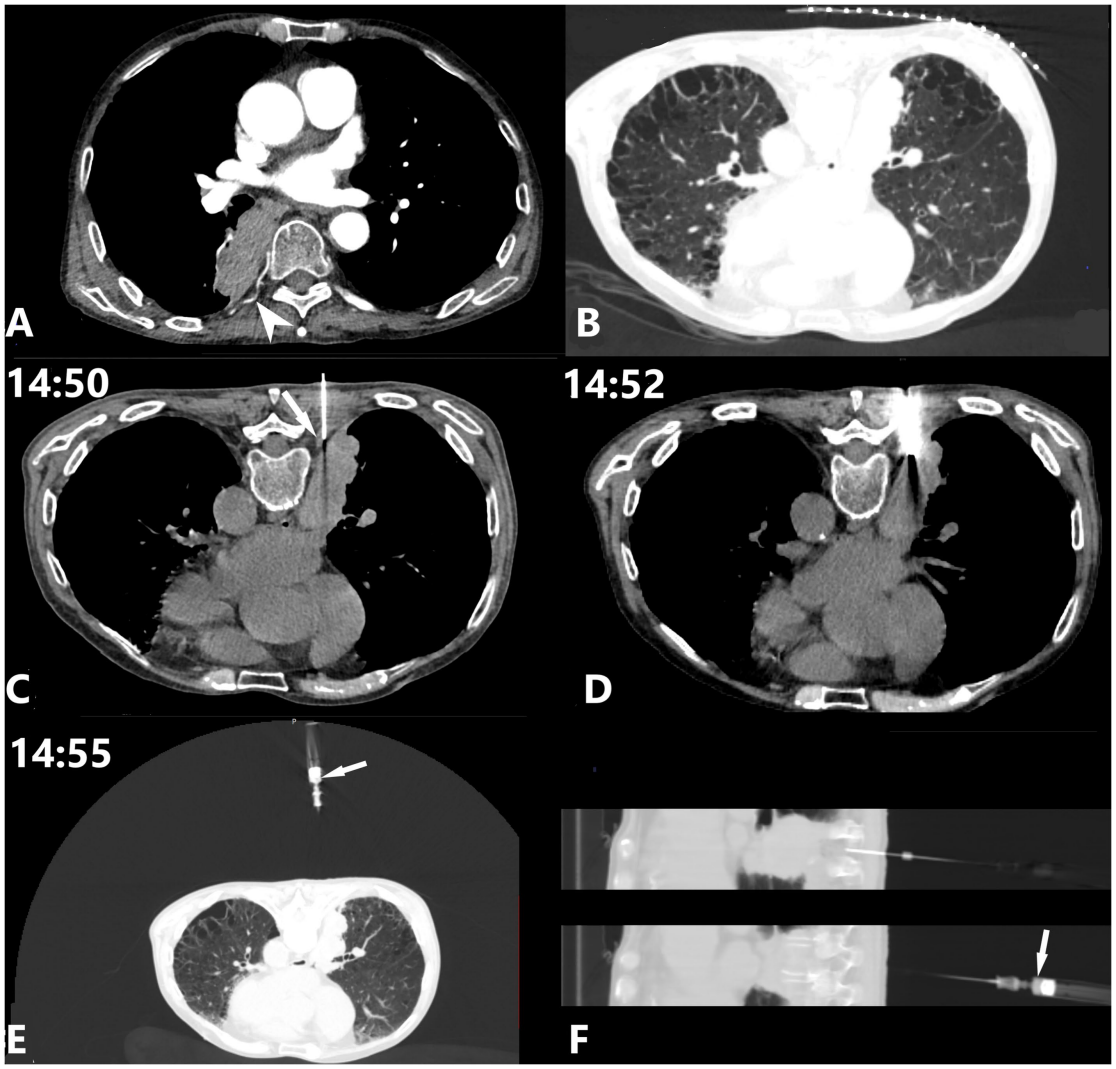
A 71-year-old male with a 10+ year history of recurrent cough and sputum production was admitted due to worsening symptoms and chest pain for 3 days. **(A,B)** Chest CT scan demonstrates emphysema with multiple bullae in both lungs, and an oval soft tissue density mass (approximately 3.0 × 6.0 cm) in the right lower lobe adjacent to the spine. An ICA (white arrowhead) courses along the posterior margin of the right lung mass. **(C)** To minimize the risk of pneumothorax, the puncture path was planned to avoid areas of emphysema and lung bullae. The coaxial puncture needle reaches the extrapleural pleura, with its medial edge adjacent to the origin segment of the ICA (white arrow). **(D)** Four tissue samples were obtained, each measuring 2.2 cm in length. **(E,F)** Bright red blood rapidly spurted into the 5 mL syringe (white arrow), with an approximate volume of 0.5 mL.

Initial contrast-enhanced chest CT revealed an oval soft tissue density mass in the right lower lobe adjacent to the spine. The scan also demonstrated severe emphysema with multiple bullae in both lungs. Notably, an ICA was observed coursing along the posterior margin of the mass (Figures 1A,B).

This case presented a challenging clinical decision due to competing risk factors: severe emphysema with multiple bullae increasing pneumothorax risk, and proximity of the target lesion to a large-caliber proximal ICA. Considering the patient’s poor pulmonary function and the relatively low reported incidence of ICA injury, the intervention team chose a posterior approach near the spine, bypassing the emphysematous areas while maintaining increased vigilance throughout the procedure.

### 2.3 Procedure description

CT-guided PTNB was performed using an 18G coaxial needle system (Monopty 1816B Bard, USA) with the patient in prone position under local anesthesia with 1% lidocaine. Initial CT scan showed the coaxial needle reaching the extrapleural space. At this point, 1 mL of 1% lidocaine was administered for local anesthesia to minimize pleural reaction during subsequent transpleural advancement. A large ICA origin segment was observed at the medial edge of the needle path (Figure 1C). The needle then reached the target mass, and four 2.2-cm-long pathological specimens were obtained (Figure 1D).

After specimen collection, needle withdrawal began. Given the proximity to the spine where the ICA is typically larger at its origin, and considering the CT image findings, the operator maintained increased vigilance for potential ICA injury. The stylet was removed, and a 5 mL syringe was attached. During slow withdrawal (approximately 1 mm/s) with slight negative pressure, 0.5 mL of bright red blood rapidly spurted into the syringe, consistent with ICA injury (Figures 1E,F).

### 2.4 Management steps

**Figure 2:**
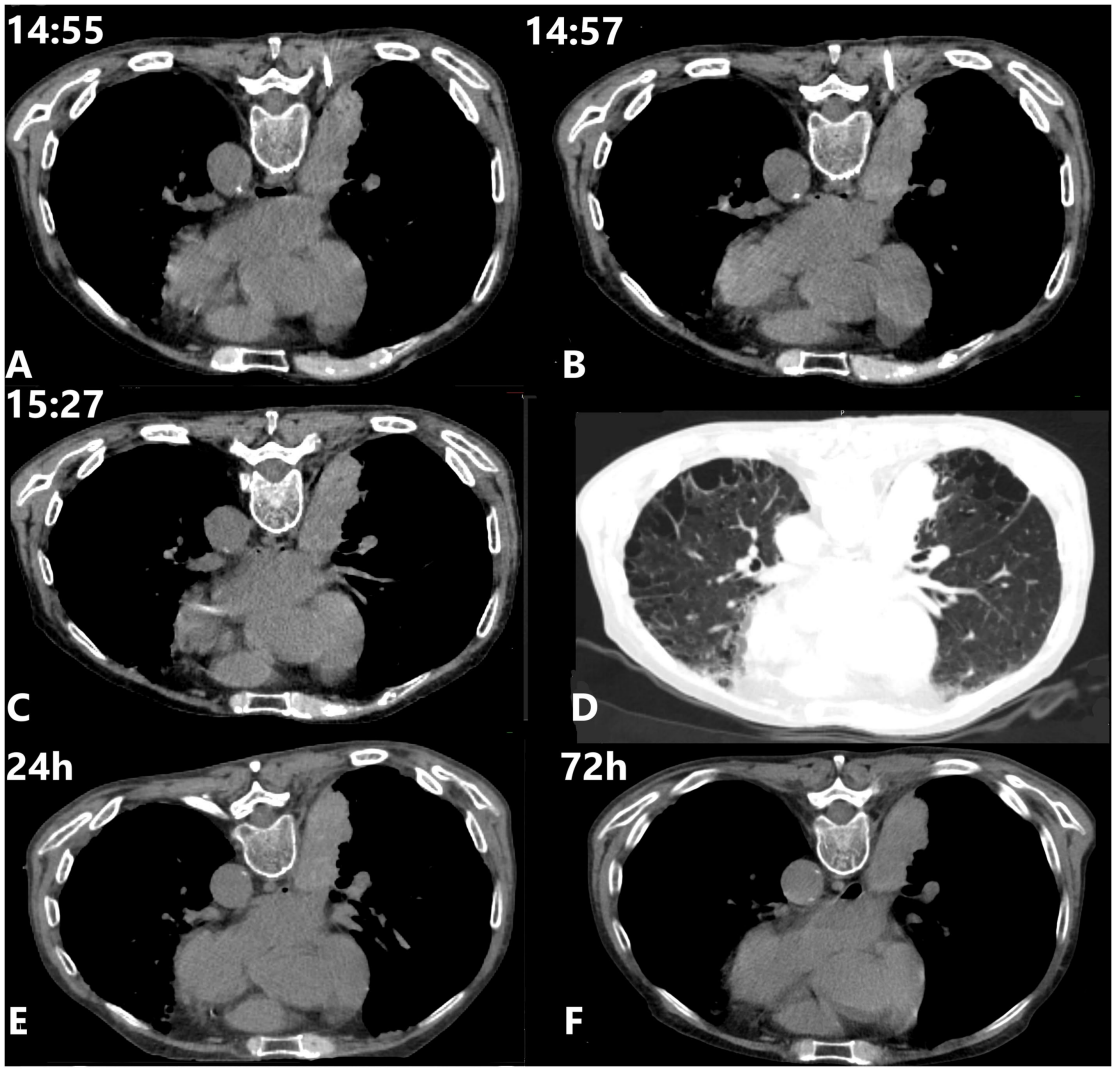
Intervention and evaluation. **(A)** Mechanical hemostasis was initiated. No visible hematoma or hemothorax was observed. The 5 mL syringe attached to the needle hub caused a slight tilt in the puncture needle. **(B)** No apparent signs of active bleeding were visible. **(C,D)** Following complete needle withdrawal, no apparent signs of active bleeding or pneumothorax were observed. **(E,F)** Follow-up CT scan images obtained at 24 and 72 h post-procedure.

The following management steps were undertaken (Figures 2A–D): (1) Immediate cessation of needle withdrawal and slight advancement of the coaxial needle by 1–2 mm. No additional blood was aspirated, and subsequent CT scan showed no visible hematoma or hemothorax; (2) Serial CT monitoring at regular intervals. The final scan showed no evidence of active bleeding after complete needle withdrawal and no pneumothorax.

### 2.5 Follow-up and outcomes

**Figure 3:**
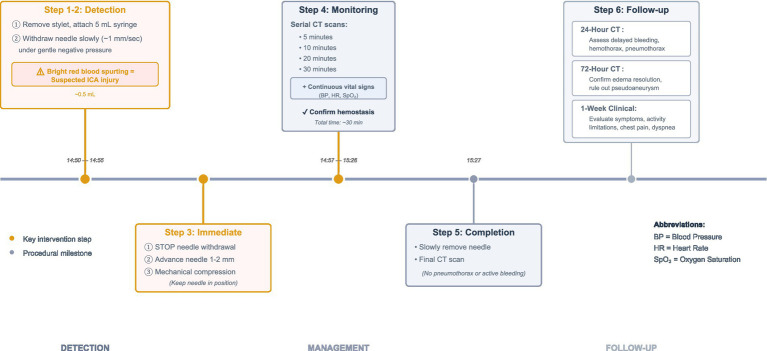
Management protocol for suspected ICA injury during PTNB.

Throughout the procedure, vital signs remained stable, and the patient reported no discomfort. CT scans performed during the hemostasis process revealed slight edema and thickening of the soft tissue surrounding the injured ICA compared to the contralateral side, with no progressive worsening or signs of active bleeding. Follow-up CT scans at 24 and 72 h post-procedure showed no evidence of pneumothorax or new hemorrhage, with gradual resolution of mild local soft tissue edema (Figures 2E,F). The procedural timeline and management sequence are summarized in Figure 3.

**Figure 4:**
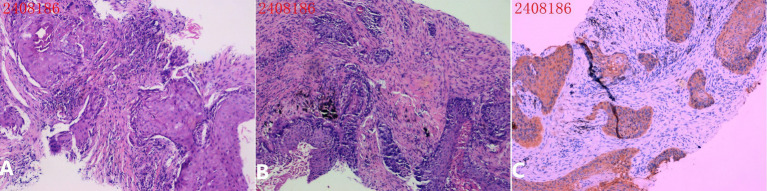
Pathological examination. **(A,B)** H&E stained pathological section: The biopsy tissue shows chronic inflammation with multiple squamous cell nests, accompanied by keratinization and incomplete keratinization. Mitotic figures are rare, and the cells appear well-differentiated. **(C)** Immunohistochemistry results: CK7 (−), TTF-1 (−), NapsinA (−), p63 (+), CK5/6 (2+), P40 (+), Ki-67 (3+, 20%).

Pathological examination confirmed well-differentiated squamous cell carcinoma (Figure 4). The patient subsequently initiated appropriate treatment. During the week-long follow-up period, the patient reported no biopsy-related pain, activity limitations, or complications.

## 3 Literature review

A systematic review of literature from 2018 to 2024 was conducted using PubMed database. Search terms included “intercostal artery injury,” “intercostal artery pseudoaneurysm,” and “hemothorax biopsy lung.” Studies reporting iatrogenic ICA injuries in thoracoabdominal procedures and hemothorax complications in PTNB were included.

### 3.1 Current management and outcomes

Literature review findings (Tables 1, 2) revealed varying management approaches for ICA injuries based on their severity. Detection timing typically ranges from intraoperative discovery to early postoperative periods, not exceeding 24 h post-procedure. Among the reviewed cases, the mortality rate was 30% (3/10), with deaths occurring between the day of injury and up to 10 days later. For surviving patients, recovery periods varied from 5 to 30 days. Currently, most ICA injuries are detected after rather than during the procedure. Management strategies depend on severity: mild cases presenting with hemothorax or local hematoma typically receive conservative treatment, while progressive or severe hemothorax may require more invasive interventions such as transcatheter arterial embolization (TAE), chest tube drainage, blood transfusion, or even thoracotomy. The consensus among reviewed studies emphasizes maintaining high suspicion for ICA injury and considering early intervention to reduce morbidity and mortality, though potential reporting bias may lead to overrepresentation of severe cases.

**Table 1:** Summary of case reports on iatrogenic ICA injury (2018–2024) (1, 2, 7–14).

References	Age/sex	Clinical context	Time to diagnosis	Presenting complication	Imaging findings	Management	Outcome
Huang et al. 2018, (1)	51/M	CT-guided lung biopsy	2 h	Massive HTX, shock	Not reported	CPR, blood transfusion, CTD	Death
Song et al. 2018, (2)	52/M	Thoracic paravertebral block	Immediate	Column-shaped hematoma	VATS findings	Conservative treatment	Recovered (7 days)
Casper et al. 2019, (7)	82/M	Thoracentesis	1 day	Respiratory distress	Large ICA pseudoaneurysm on CTA	TAE, CTD	Recovered (5 days)
Bozhchenko et al. 2019, (9)	Unknown	Pleural puncture	Unknown	Unknown	Unknown	Unknown	Death
Berry 2020, (8)	47/M	Paracentesis	12 h	Hypotension, tachycardia	Massive HTX on CT	TAE, CTD	Death (10 days)
Laugsand and Xanthoulis 2020, (10)	74/M	Pleural drain placement	4 h	Massive HTX	HTX on CXR	Thoracotomy	Recovered (8 days)
Park and Lee 2021, (11)	59/F	Pleural catheter removal	15 min	Hypotension, tachycardia	Massive HTX on CT	TAE, CTD	Recovered (30 days)
Chou and Hsieh 2022, (12)	70/F	Thoracentesis	1 day	Dyspnea	ICA extravasation on CTA	TAE, CTD	Recovered
Warhadpande et al. 2023, (13)	69/M	Cryoablation	Intraoperative	Hypotension, ventilation difficulties	Large HTX on CT	TAE, CTD	Recovered (7 days)
Kakinoki et al. 2023, (14)	56/F	Percutaneous nephrolithotripsy	2 days	Bleeding shock	Not reported	TAE	Recovered (30 days)

M, Male; F, Female; HTX, Hemothorax; CPR, Cardiopulmonary Resuscitation; CTD, Chest Tube Drainage; VATS, Video-Assisted Thoracoscopic Surgery; ICA, Intercostal Artery; CTA, Computed Tomography Angiography; TAE, Transcatheter Arterial Embolization; CT, Computed Tomography; CXR, Chest X-Ray.

Cases are listed in chronological order.

Bozhchenko, 2019 is a Russian language publication with English abstract.

**Table 2:** Incidence and management of hemothorax in CT-guided PTNB: a review of original research (2018–2024) (15–21).

Study	Sample size	Patient demographics	HTX incidence	Management of HTX	Outcome
Jeon et al. 2018, (15)	243	66.5 ± 11.3 years, 64.8% male	1.6% (4/243)	Oxygen therapy, observation	Recovery (all cases)
Huang et al. 2018, (16)	72	65.9 years, 54.2% male	1.4% (1/72)	Not reported	Death (1 case)
Watane et al. 2019, (17)	166	59 (25–87) years, 54.2% male	3.0% (5/166)	Not reported	Death (1 case)
Ahn and Jang 2019, (18)	248	68.2 ± 12.5 years, 70.2% male	0.8% (2/248)	Chest tube drainage	Not reported
Gershman et al. 2022, (19)	91	71.1 years, 58.2% male	1.1% (1/91)	Chest tube drainage	Recovery
Chen et al. 2023, (20)	41	66 (58–76) years, 46.3% male	4.9% (2/41)	Not reported	Not reported
Ezenagu et al. 2024, (21)	1,063	66 (21–92) years, 50.5% male	0.38% (4/1063)	Not reported	Recovery (all cases)

HTX, Hemothorax; CT, Computed Tomography.

## 4 Discussion

This improvised intraoperative management decision was formulated based on two factors: (1) preoperative imaging demonstrating ICA proximity and intraoperative CT findings suggesting potential complications, and (2) adaptation of detection techniques from CT-guided percutaneous transhepatic biliary drainage, extended to include therapeutic needle repositioning for hemostasis. In biliary procedures, when a coaxial needle inadvertently penetrates through the bile duct wall, the established protocol involves removing the stylet, attaching a 5 mL syringe to the needle hub, and slowly withdrawing the needle under slight negative pressure. Bile aspiration confirms when the needlepoint is within the duct, thus facilitating subsequent guidewire insertion. This diagnostic approach parallels established clinical practice protocols, where indirect procedural evidence guides decision-making. A comparable example is the routine use of pre-injection aspiration to prevent inadvertent intravascular lidocaine administration and its associated adverse effects—a standard safety measure based on aspiration results rather than direct vascular visualization. The coaxial needle appeared to provide dual hemostatic functions: (1) mechanical compression of the injured vessel, promoting vasoconstriction and thrombus formation, and (2) coagulation stimulation through foreign body-induced platelet aggregation and fibrin formation (3, 4).

Recent studies report hemothorax incidence rates ranging from 0.38 to 4.9%, higher than earlier reported rates (5). In 2019, Tamburini et al. reported that among 30 cases of ICA injuries managed with TAE at two institutions over 16 months, iatrogenic injuries accounted for 37% (11/30) (6). When faced with ICA injury during PTNB, outcomes can vary widely, ranging from patient recovery with conservative treatment to rapid deterioration and death (1, 7, 8). This case suggests a potential stepwise approach for managing suspected ICA injury that might reduce treatment time and minimize complications while decreasing the need for more invasive interventions. In resource-limited settings where interventional radiology services may not be immediately available, having alternative management options could be valuable.

Several important limitations must be acknowledged. The diagnosis of ICA injury was based primarily on clinical observation and imaging findings, without angiographic confirmation. Additionally, as a single case report representing the lowest level of clinical evidence, this observation should be considered hypothesis-generating and requires further validation. Given that the rarity of this complication limits opportunities for single-center validation, publication of this case report serves to enable other operators to evaluate this approach when similar circumstances arise, thereby facilitating the necessary collaborative validation. The applicability of this method may be influenced by various factors, including vessel size, injury severity, and operator experience in detecting and responding to injury signs.

### 4.1 Patient perspective

The patient expressed satisfaction with the procedure and appreciated the prompt management of the complication. He reported minimal discomfort during and after the procedure, and was particularly relieved to have avoided both pneumothorax and additional invasive interventions such as chest tube placement.

## 5 Conclusion

This case describes a cautious approach for managing suspected ICA injury during PTNB that may serve as one potential management option for consideration in similar high-risk scenarios.

## Data Availability

The data analyzed in this study is subject to the following licenses/restrictions: Patient clinical data is protected under medical privacy regulations and institutional policies. Raw clinical data cannot be shared publicly due to patient confidentiality requirements. The literature review data are publicly available through the cited references. Summary data and analysis results are presented within the manuscript tables and text. Requests to access these datasets should be directed to Hao Zhang, plutoye_7@hotmail.com.

## References

[1] Huang WM Lin HC Chen CH Chen CW Wang CH Huang CY . Massive hemothorax after computed tomography-guided lung tumor biopsy: an unusual but disastrous complication. Thorac Cancer. (2018) 9:892–6. doi: 10.1111/1759-7714.12769, PMID: 29791072 PMC6026619

[2] Song L Zhou Y Huang D. Inadvertent posterior intercostal artery puncture and haemorrhage after ultrasound-guided thoracic paravertebral block: a case report. BMC Anesthesiol. (2018) 18:196. doi: 10.1186/s12871-018-0667-5, PMID: 30577774 PMC6303859

[3] Kizhakkedathu JN Conway EM. Biomaterial and cellular implants: foreign surfaces where immunity and coagulation meet. Blood. (2022) 139:1987–98. doi: 10.1182/blood.2020007209, PMID: 34415324

[4] Al-Amer OM. The role of thrombin in haemostasis. Blood Coagul Fibrinolysis. (2022) 33:145–8. doi: 10.1097/MBC.0000000000001130, PMID: 35239615

[5] Tomiyama N Yasuhara Y Nakajima Y Adachi S Arai Y Kusumoto M . CT-guided needle biopsy of lung lesions: a survey of severe complication based on 9783 biopsies in Japan. Eur J Radiol. (2006) 59:60–4. doi: 10.1016/j.ejrad.2006.02.001, PMID: 16530369

[6] Tamburini N Carriel N Cavallesco G Molins L Galeotti R Guzmán R . Technical results, clinical efficacy and predictors of outcome of intercostal arteries embolization for hemothorax: a two-institutions' experience. J Thorac Dis. (2019) 11:4693–9. doi: 10.21037/jtd.2019.10.27, PMID: 31903258 PMC6940252

[7] Casper KP Sanchirico PJ Pfeiffer DC. Intercostal artery pseudoaneurysm following thoracentesis: multi-modal imaging and treatment. BMC Med Imaging. (2019) 19:31. doi: 10.1186/s12880-019-0333-5, PMID: 31029094 PMC6487039

[8] Berry AC. Hemorrhagic complications of paracentesis: aberrant anatomy versus aberrant technique. Cureus. (2020) 12:e8827. doi: 10.7759/cureus.8827, PMID: 32742840 PMC7384730

[9] Bozhchenko AP Tolmachev IA Belykh AN. Nastuplenie letal'nogo iskhoda vsledstvie iatrogennogo povrezhdeniia vetvi mezhrebernoĭ arterii pri provedenii plevral'noĭ punktsii [occurrence of a lethal outcome due to iatrogenic damage of an intercostal vessel during a pleural puncture procedure]. Sud-Med Ekspert. (2019) 62:58–62. doi: 10.17116/sudmed20196206158 31825335

[10] Laugsand EA Xanthoulis A. Management of a life-threatening intercostal artery bleeding, difficult to visualize in open surgery: a case report. J Surg Case Rep. (2020) 2020:rjaa444. doi: 10.1093/jscr/rjaa444, PMID: 33154815 PMC7602520

[11] Park C Lee J. Massive hemothorax due to intercostal arterial bleeding after percutaneous catheter removal in a multiple-trauma patient: a case report. World J Clin Cases. (2021) 9:9942–7. doi: 10.12998/wjcc.v9.i32.9942, PMID: 34877334 PMC8610904

[12] Chou CH Hsieh HJ. Haemothorax due to intercostal artery injury after thoracentesis. Respirol Case Rep. (2022) 10:e0950. Published 2022 Apr 15. doi: 10.1002/rcr2.950, PMID: 35441034 PMC9011361

[13] Warhadpande S Liles A Kirkpatrick D. Intercostal artery laceration after adrenal mass cryoablation. Semin Intervent Radiol. (2023) 40:286–9. doi: 10.1055/s-0043-1769766, PMID: 37484442 PMC10359126

[14] Kakinoki H Yamaguchi Y Yukimoto M Kakinoki Y Udo K Tobu S . A case of bleeding shock induced by injury of the intercostal artery following percutaneous nephrolithotripsy. IJU Case Rep. (2023) 7:18–21. doi: 10.1002/iju5.12657, PMID: 38173459 PMC10758889

[15] Jeon MC Kim JO Jung SS Park HS Lee JE Moon JY . CT-guided percutaneous transthoracic needle biopsy using the additional laser guidance system by a pulmonologist with 2 years of experience in CT-guided percutaneous transthoracic needle biopsy. Tuberc Respir Dis (Seoul). (2018) 81:330–8. doi: 10.4046/trd.2017.0123, PMID: 29926547 PMC6148095

[16] Huang WM Chen CH Liang SH Huang CY Cheng SM Sheu CY . Multiplanar reconstruction technique for difficult computed tomography-guided lung biopsy: improved accuracy and safety. Thorac Cancer. (2018) 9:1333–7. doi: 10.1111/1759-7714.12835, PMID: 30094947 PMC6166063

[17] Watane GV Hammer MM Barile MF. CT-guided Core-needle biopsy of the lung is safe and more effective than fine-needle aspiration biopsy in patients with hematologic malignancies. Radiol Cardiothorac Imaging. (2019) 1:e180030. doi: 10.1148/ryct.2019180030, PMID: 33778526 PMC7977995

[18] Ahn JH Jang JG. Initial experience in CT-guided percutaneous transthoracic needle biopsy of lung lesions performed by a pulmonologist. J Clin Med. (2019) 8:8. doi: 10.3390/jcm8060821, PMID: 31181794 PMC6616495

[19] Gershman E Vaynshteyn I Freidkin L Pertzov B Rosengarten D Kramer MR. Marked safety and high diagnostic yield of freehand ultrasound-guided core-needle biopsies performed by pulmonologists. Thorac Cancer. (2022) 13:1577–82. doi: 10.1111/1759-7714.14413, PMID: 35474608 PMC9161330

[20] Chen LC Yang SM Malwade S Chang HC Chang LK Chung WY . Cone-beam computed-tomography-derived augmented fluoroscopy-guided biopsy for peripheral pulmonary nodules in a hybrid operating room: a case series. Diagnostics (Basel). (2023) 13:1055. Published 2023 Mar 10. doi: 10.3390/diagnostics13061055, PMID: 36980363 PMC10047390

[21] Ezenagu OC Gabriel GE Saha SP. Computed tomography (CT)-guided needle biopsy of lung lesions: a single center experience. Healthcare (Basel). (2024) 12:1260. Published 2024 Jun 25. doi: 10.3390/healthcare12131260, PMID: 38998796 PMC11240914

